# The Emerging Role of Artificial Intelligence in the Assessment of Valvular Heart Disease with Cardiac Imaging

**DOI:** 10.1007/s11886-025-02344-2

**Published:** 2026-01-25

**Authors:** Cory Sejo, Michael Randazzo, Roberto Lang, Jeremy Slivnick

**Affiliations:** The University of Chicago Medical Center, Section of Cardiology, 5841 S Maryland Ave, MC 6080, Chicago, IL 60637 USA

**Keywords:** Artificial intelligence, Deep learning, Machine learning, Valvular heart disease, Cardiac imaging

## Abstract

**Purpose of Review:**

This review summarizes current applications of artificial intelligence (AI) in multimodality cardiac imaging for the evaluation of valvular heart disease (VHD).

**Recent Findings:**

The prevalence of VHD continues to rise, placing increasing demands on cardiovascular imaging and longitudinal management. AI systems have been applied across echocardiography, cardiac computed tomography (CCT), and cardiac magnetic resonance (CMR) to automate image classification, segmentation, disease detection, and severity assessment. The most mature AI models have centered on transthoracic echocardiography (TTE), where deep learning (DL) frameworks enable whole-study interpretation and preliminary report generation. Applications in CCT and CMR remain in earlier stages but show promise for segmentation, tissue characterization, and pre-procedural planning.

**Summary:**

AI has the potential to enhance the accuracy, reproducibility, and efficiency of imaging-based VHD assessment. Key challenges remain around generalizability, transparency, and clinical integration. Multidisciplinary collaboration is essential to ensure that AI complements, rather than replaces, human expertise.

## Introduction

With advancing life expectancy, the burden of valvular heart disease (VHD) has risen substantially, with total mortality more than doubling over the past three decades [1]. Parallel advances in surgical and transcatheter therapies have broadened access to valve interventions for higher-risk patients, heightening the need for accurate imaging-based detection, quantification, and prognostication [2–6].

Transthoracic echocardiography (TTE) remains the frontline imaging tool for VHD due to its accessibility and low cost. Comprehensive morphologic and hemodynamic evaluation through two-dimensional (2D), three-dimensional (3D), and Doppler imaging informs diagnosis, surveillance, and treatment planning [6, 7]. However, interpretation is constrained by growing imaging volumes, workforce shortages, and inter-observer variability [8–11].

Complementary modalities, such as transesophageal echocardiography (TEE), cardiac magnetic resonance (CMR), or cardiac computed tomography (CCT), provide additional structural and functional information [12], but are resource-intensive and often require laborious manual post-processing [13–15].

These challenges underscore the need for scalable, automated imaging tools. AI-based systems can improve accuracy, efficiency, and reproducibility across imaging modalities. Within echocardiography, CCT, and CMR, AI tools streamline workflows, enhance quantitative analysis, and expand access [12, 16–18]. This review outlines the current and emerging role of AI in multimodality VHD imaging, highlights knowledge gaps, and proposes future directions.

## Defining Artificial Intelligence

**Fig. 1:**
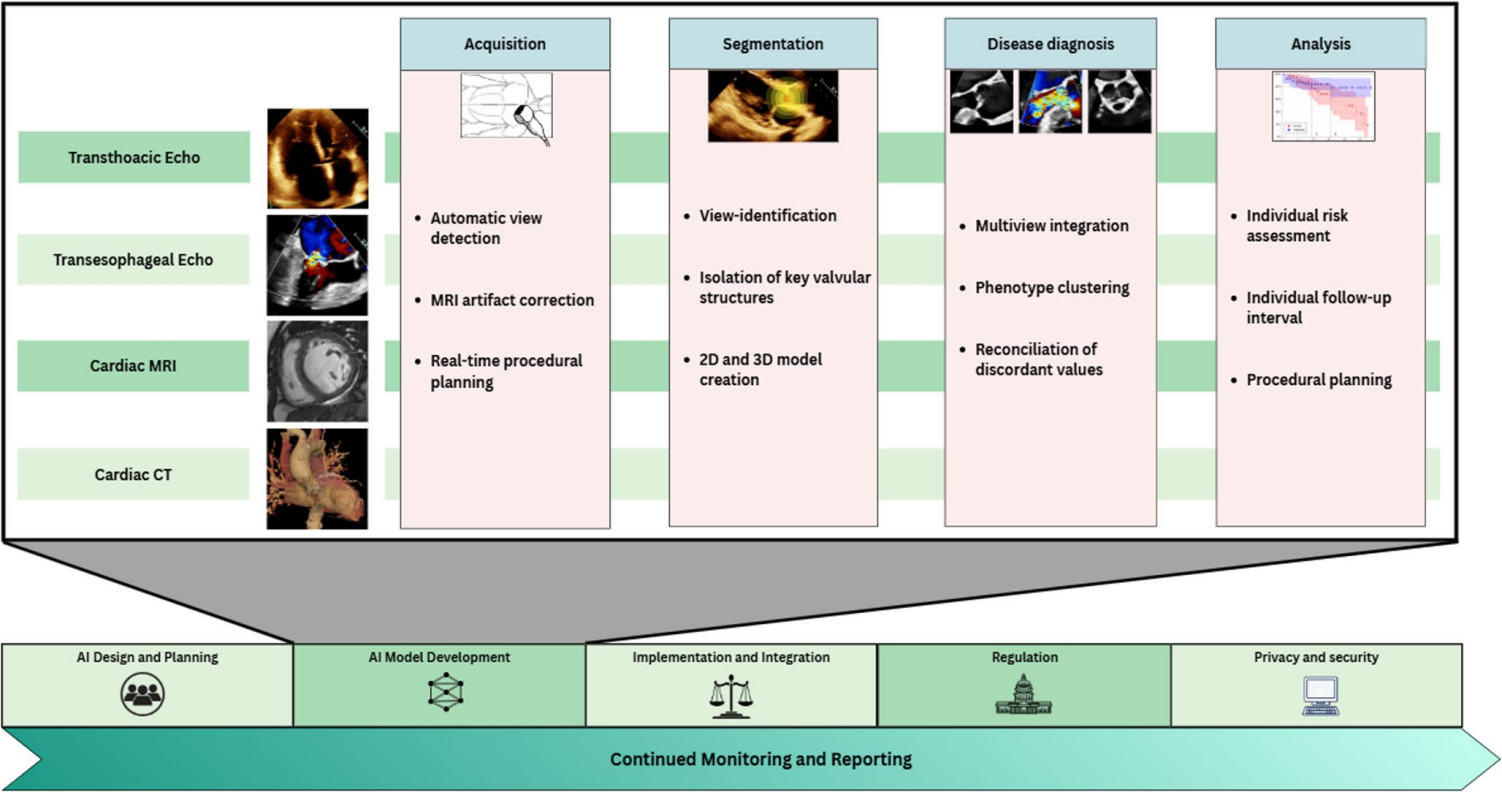
Steps for responsible AI model development and implementation. Figure created in Canva (https://www.canva.com). MRI = magnetic resonance imaging; CT = computed tomography. 2D = two-dimensional; 3D = three-dimensional; echo = echocardiography

Artificial intelligence broadly refers to computational systems that emulate human cognitive functions such as perception, learning, and decision-making [19, 20]. Within AI, machine learning (ML) encompasses algorithms that identify patterns and learn predictive relationships from data, while deep learning (DL) is a subtype of ML that uses layered neural networks to autonomously extract hierarchical features from raw images without explicit programming [19, 20]. ML is trained in a supervised (with labeled data) or unsupervised (detecting hidden patterns without labels) manner [19, 20]. These approaches form the basis for most current VHD algorithms. In cardiac imaging, AI applications extend across the continuum of care, including optimizing scan planning and acquisition, view recognition, structure segmentation, quantitative measurement, disease detection, and analysis (risk stratification and procedural guidance) (Fig. 1) [15, 21–24].

### Entire-study Assessment by AI

Before turning to valve-specific applications, it is helpful to review comprehensive frameworks that analyze entire echocardiographic studies. These systems emulate human interpretation by integrating information across multiple views and measurements to generate exam-level outputs. Although early AI models to assess VHD relied on limited views, often to address single tasks [25–27], recent DL models aim to emulate full-study interpretation. These include tasks ranging from automated view identification, automated measurements, and report generation.

One such model, DELINEATE, introduced a multiview DL system trained on over 70,000 TTEs from two centers to evaluate regurgitant lesions. This framework automatically detects and analyzes relevant doppler echocardiographic views to predict the presence and severity of regurgitant lesions. Originally developed for mitral regurgitation, later iterations (DELINEATE-Regurgitation) extended to aortic and tricuspid regurgitation, achieving strong agreement with expert readers (weighted κ ≈ 0.7) [28, 29]. Importantly, this model improved prediction of MR progression, illustrating AI’s potential for diagnosis and longitudinal risk assessment [28].

Around the same time, Yang et al. developed a supervised DL model trained on ~ 1,600 echocardiographic studies from five hospitals using limited quantitative and semi-quantitative parameters to detect and grade multiple valvular lesions, including aortic regurgitation (AR), aortic stenosis (AS), mitral regurgitation (MR), and mitral stenosis (MS) [30]. This model achieved excellent diagnostic accuracy, with area under the receiver operating characteristic (AUROC) values of 0.88–0.99 across lesions, and inter-reader agreement comparable to expert-expert variability. This model’s prospective validation of ~ 1,300 TTEs was a major strength [30]. However, reliance on Doppler-based inputs limited precision for complex lesions requiring integration of non-Doppler views.

Simultaneous to Yang et al., a supervised DL algorithm called *Us2.ai* was developed to accurately measure over 45 TTE parameters and generate a preliminary report. This FDA-approved system demonstrated excellent agreement with human measurements [31, 32] and has been validated in AS [33], MR [34], and TR [35] with high accuracy. Notably, it has entered clinical use, improving efficiency, image quality, and reducing human fatigue [36].

Following *Us2.ai*, the PanEcho framework developed a fully automated two dimensional (2D) TTE image analysis and reporting platform [22]. Trained on over 32,000 studies from multiple centers, it identifies 2D and Doppler echocardiographic views and generates unified study-level interpretations approximating the comprehensive analysis of human echocardiographers. It was extensively validated internally and externally in a diverse cohort (New England, Budapest, and two sites in California), including on simplified scans from novice scanners [22]. As an open-source platform, PanEcho serves as a valuable benchmark for transparent and reproducible AI echocardiography research.

The EchoNet family contributed task-specific models: EchoNet-LVH for detecting left ventricular (LV) hypertrophic phenotypes [37], EchoNet-Dynamic for automated ejection fraction and wall motion [38], and EchoNet-Measurements, which was trained on over 150,000 TTEs to automate 18 standard TTE measurements [21]. The latter model demonstrated high agreement with sonographer-derived values (mean coverage probability ≈ 0.80–0.84) and could serve as a foundation for AI-assisted measurements and reporting.

A recently developed vision-language model called EchoPrime evaluated entire TTE studies similar to PanEcho [39]. Trained using contrastive (unsupervised) learning on over 275,000 TTEs at a single institution, it aligns unlabeled images with text reports to generate draft summaries. Like PanEcho, EchoPrime is open-source and designed for multi-view, multi-task learning, offering a flexible framework for cross-domain echocardiographic analysis.

Together, these frameworks represent a paradigm shift in echocardiographic AI from narrow, single-task algorithms toward comprehensive, study-level systems that can integrate multi-view data and emulate expert reader workflow. These models are the culmination of many systems described in the subsequent valve-specific sections.

## Aortic Valve: *Aortic stenosis*

### Echocardiography in Aortic Stenosis

Aortic stenosis (AS) is the most common form of valvular heart disease in developed countries, with increasing incidence among aging populations and representing the leading cause of VHD-related mortality [40, 41]. AS is primarily driven by age-related fibrocalcific remodeling causing valvular narrowing and resultant flow obstruction. Echocardiography remains the cornerstone of assessment of valve morphology, left ventricular (LV) size and function, and Doppler-based hemodynamic gradients [6, 7]. However, the multifactorial nature of AS, requiring integration of multiple imaging parameters and serial follow-up, makes it an ideal target for AI applications that enhance reproducibility and efficiency.

Early DL models have shown strong performance in detecting severe AS from limited echocardiographic views. For example, self-supervised networks trained on single non-Doppler parasternal long-axis (PLAX) images have achieved AUROC of 0.98 for severe AS detection [25]. Other supervised models using similarly restricted datasets have reported modest accuracy (AUROC ~ 0.75) [26]. Simplified systems have created novel biomarkers such as the Digital AS Severity index (DASSi), which was trained on 58,000 non-Doppler PLAX images to predict both AS severity and disease progression [42]. These 2D-only systems may have particular utility in low resource settings limited to non-Doppler POCUS examinations.

A novel decision support algorithm (AI-DSA) was effective at detecting low-flow, low-gradient (LFLG) AS and predicting outcomes by integrating nonclassic parameters like LV structural data [43]. Previously described fully comprehensive frameworks including PanEcho, EchoPrime, and *Us2.AI*, extend beyond single-view models and demonstrate exceptional accuracy for the detection of moderate-to-severe AS as compared to expert readers (AUC 0.96-1.00) (Table 1) [22, 30, 33, 39].

Transesophageal echocardiography may be pursued when TTE visualization of valvular structure or Doppler jets is suboptimal, or for procedural planning. Small studies utilizing both DL and ML software in the peri-procedural segmentation of key aortic valve structures, such as the aortic annulus and root, have shown high accuracy compared to CCT-derived measurements and can improve efficiency [44–46]. In stratifying AS disease severity, categorizing AS phenotypes, and predicting disease progression, AI algorithms can personalize management and reduce unnecessary follow-up TTEs by 13–49% [23, 42, 47, 48].

### CCT and CMR in Aortic Stenosis

When TTE findings are uncertain or discordant, such as in LFLG AS, further downstream testing with CMR or CCT may be required for diagnosis and risk stratification. CCT plays an increasingly central role by quantifying aortic valve calcium, performing precise anatomic measurements, and enabling coronary evaluation for procedural planning [6, 7]. Despite their importance, CCT-derived measurements are time-consuming to perform.

DL algorithms have demonstrated excellent performance in automating calcium quantification, even in non-dedicated, low-dose chest CTs [49–51]. These methods support opportunistic screening and reduce manual analysis time. Several small but promising DL studies have also shown high accuracy in key pre-procedural measurements of the aortic annulus and root [24, 52, 53]. One such framework, *CardioVision*, generates automated 3D reconstruction of the aortic root, quantifies calcium burden and spatial distribution, and offers a potentially powerful adjunct for transcatheter valve planning [53, 54].

Although CMR remains a third-line modality after TTE and CT for routine AS assessment, its strength lies in tissue characterization, enabling detection of subclinical interstitial fibrosis with prognostic implications [55, 56]. Novel ML algorithms using CMR can identify interstitial fibrosis and adverse remodeling, which is predictive of adverse outcomes after transcatheter aortic valve implantation (TAVI) and may support patient selection for intervention [57].

## Aortic Valve: *Aortic Regurgitation*

### Echocardiography in Aortic Regurgitation

Aortic regurgitation (AR) accounted for approximately 3.3% of VHD-related deaths in the U.S. between 1999 and 2020 [41]. AR arises from either intrinsic leaflet disruption or aortic root pathology, and accurate characterization of both is critical for clinical management [58]. TTE remains the first-line modality; however, grading AR severity is limited by inter-reader variability, eccentric jets, or suboptimal image quality [58, 59]. TEE provides complementary evaluation when visualization of the aortic valve or root is inadequate, particularly for procedural or surgical planning [58].

AI systems can enhance standardization and reproducibility in AR quantification by integrating information across the entire echocardiographic study. The previously described DELINEATE-Regurgitation model found that multiview detection of AR performed better than single apical Doppler views in identifying moderate-or-greater regurgitation (AUC 0.980–0.992) [28]. Similarly, Yang et al., PanEcho, and EchoPrime displayed high accuracy in detecting AR with AUCs of 0.85–0.98 **(**Table 1) [22, 30, 39]. These comprehensive, study-level approaches emulate the integrative, multiparametric assessment of expert echocardiographers and are recommended by current guidelines [58].

Despite these advances, several limitations remain. Most algorithms are trained on 2D echocardiography, whereas 3D color Doppler imaging is now increasingly used for quantifying regurgitant volume and vena contracta area, which has not yet been widely incorporated [60]. Additionally, few AI frameworks integrate TEE or POCUS data despite their frequent clinical use for emergent and perioperative evaluations.

### CMR and CTT in Aortic Regurgitation

CMR serves as a valuable adjunct to echocardiography when image quality is inadequate or when quantification discrepancies arise. It enables direct regurgitant volume measurement, identification of the AR mechanism, and assessment of myocardial fibrosis or remodeling, which are factors that can guide treatment timing [58]. Using an unsupervised learning approach, Malahfji et al. analyzed CMR data from almost 1,000 patients with moderate-to-severe AR across four centers and identified leaflet morphology, sex, and LV scar as phenotypic predictors of outcomes [61, 62]. These findings suggest a future role for AI-enhanced CMR in risk stratification and timing of intervention.

CCT plays a more limited but complementary role, particularly for assessing the aortic root and ascending aorta during pre-operative planning [24, 52, 53, 63].

### Mitral Valve Disease

Mitral valve disease remains a major contributor to global cardiovascular morbidity, encompassing a wide spectrum from rheumatic and degenerative MS to primary and secondary MR [1]. TTE serves as the cornerstone for diagnosis, grading, and serial follow-up, while TEE provides complementary high-resolution assessment of leaflet morphology, the subvalvular apparatus, and suitability for intervention. CMR can play a role in the evaluation of MR through tissue characterization and in quantifying regurgitation, but CCT and CMR for the evaluation of MS is limited.

### Echocardiography, CMR, and CCT in Mitral Stenosis

Mitral stenosis most commonly arises from rheumatic fever in developing countries and degenerative diseases in older populations [64]. Evaluation requires integration of anatomic and hemodynamic parameters across multiple echocardiographic views, such as mitral valve area, pressure half-time, and mean gradient, which may be time consuming and prone to inter-observe variability. AI can streamline these analyses by automating key measurements. DL algorithms in particular have accurately measured pressure half-time and identified MS with near-perfect classification accuracy (AUC ≈ 0.99) using limited input data [30]. Broader, whole-study systems like PanEcho and EchoPrime have demonstrated similarly high accuracy for detecting MS across diverse imaging datasets (AUC of 0.92-1.00) [22, 39], suggesting a role for automated case screening and quantification.

While these tools can enhance reproducibility, they remain limited in delineating detailed valvular morphology. Emerging segmentation frameworks using 2D and 3D TEE can automatically map the mitral annulus and leaflet motion, improving consistency in planimetry and facilitating preprocedural planning [65–67]. Continued refinement of these models may further reduce operator dependency and enable real-time quantitative guidance for interventions.

## Mitral Regurgitation

### Echocardiography in Mitral Regurgitation

Mitral regurgitation is among the most prevalent valvular lesions and carries significant prognostic implications. Accurate quantification of MR severity and assessment of LV remodeling are crucial for determining timing of intervention and predicting outcomes. The mitral apparatus is anatomically complex, comprising the annulus, leaflets, chordae tendineae, and papillary muscles, all of which must function synchronously. Because of this complexity, MR evaluation requires integration of multiple imaging parameters and views, often leading to inter-reader variability.

DL frameworks have demonstrated strong potential to enhance reproducibility and diagnostic consistency. Early single-view models, such as those using PLAX or apical 4 chamber (A4C) views, identified rheumatic MR with high accuracy (AUROC 0.84) [68], and others replicated quantitative techniques such as the proximal isovelocity surface area (PISA) measurement using DL automation [27, 69]. Yet, these systems were inherently limited by their inability to synthesize multiparametric data.

The previously described DELINEATE-Regurgitation framework represented a major advance by integrating multiview Doppler inputs from thousands of complete TTE studies to classify MR severity. The model achieved strong agreement with expert readers and, importantly, stratified MR progression risk with a hazard ratio of 4.1 despite controlling for other clinical factors [28, 29]. This demonstrates that AI models can extend beyond static classification to dynamic risk prediction. However, because this and similar models relied primarily on Doppler data, they did not capture morphological information, which remains an area for future refinement [6, 70].

Broader, study-level models such as PanEcho, EchoPrime, *Us2.ai*, and Yang et al.’s multivew system have further advanced MR evaluation by integrating information across entire echocardiographic studies. These frameworks demonstrated high diagnostic performance for detecting moderate-or-greater MR (AUC 0.88–0.95) [22, 30, 34, 39], mirroring the comprehensive synthesis performed by expert readers and aligning with guideline-recommended multiparametric assessment [6]. They hold promise for automated preliminary reporting and workflow triage in high-volume echo laboratories.

Beyond detection, ML approaches using standard TTE parameters have identified distinct MR phenotypes with differing postoperative outcomes. For example, an explainable ML model integrating features such as E/e’ ratio, PISA, septal wall thickness, and regurgitant orifice area successfully stratified patients with primary MR into clusters predictive of long-term survival [71]. Such clustering frameworks may ultimately complement guidelines-based severity grading by incorporating subclinical imaging features reflective of myocardial and valvular remodeling.

### CMR and CCT in Mitral Regurgitation

CMR provides an important adjunct when echocardiographic findings are inconclusive. Advanced 4D flow MRI coupled with ML-based segmentation enables fully automated quantification of regurgitant volume and simultaneous tracking biventricular function, enhancing reproducibility over manual workflows [72, 73]. CCT, while limited for functional assessment, plays an expanding role in procedural planning by offering precise 3D modeling of the mitral annulus, leaflet geometry, and coronary relationship to guide transcatheter intervention [74].

Collectively, these multimodality and AI-driven approaches are moving MR evaluation toward a more standardized, quantitative, and predictive framework. While human expertise remains indispensable for integrating imaging findings with clinical context, automated systems are poised to improve consistency, streamline workflow, and refine patient selection for intervention.

### Tricuspid Valve

Tricuspid valve (TV) disease is increasingly recognized as a major contributor to morbidity and mortality among patients with VHD [75]. Once regarded as secondary to left-sided lesions, TR is now understood to carry an independent adverse prognosis, with even mild TR associated with worse long-term survival [76–78]. The recent expansion of transcatheter tricuspid interventions has heightened interest in accurate and reproducible imaging of TV anatomy and function [2, 3].

### Echocardiography in Tricuspid Regurgitation

TTE remains the first-line modality for evaluation of TR, providing hemodynamic assessment and real-time procedural guidance. However, accurate quantification of TR can be challenging due to the complex geometry of the right heart and the variability of Doppler signal alignment. 3D echocardiography improves visualization of leaflet morphology and annular dimensions but remains time-intensive and operator-dependent.

DL systems have demonstrated strong potential to enhance reproducibility in TR assessment. The EchoNet-TR model analyzed entire TTE studies to automatically identify A4C color Doppler views and detect severe TR with high accuracy (AUC ≈ 0.98) [79]. Similarly, the DELINEATE-Regurgitation framework integrated multiview Doppler data to classify moderate-or-greater TR, achieving AUCs of 0.95–0.98 [28]. PanEcho, EchoPrime, and *Us2.ai* extend this capability to full-study analysis, offering integrated grading of TR severity across multiple echocardiographic views with high accuracy (AUC ≈ 0.90–0.95)(Table 1) [22, 39, 80]. Collectively, these approaches emulate expert echocardiographic evaluation and may support early screening and serial follow-up.

Beyond grading, ML has been applied to identify TR phenotypes and predict clinical outcomes. Unsupervised clustering of patients with significant TR has revealed distinct subgroups with differing hemodynamic profiles and three-year mortality risk, highlighting the potential of data-driven classification to complement conventional assessment [77, 80, 81]. Supervised ML algorithms have also been used to refine RV systolic pressure estimation and optimize patient selection for transcatheter repair [82].

### CMR and CCT in Tricuspid Regurgitation

CMR plays an important complementary role for quantification of severity when echocardiographic results are discordant or when quantitative assessment of RV volumes and function are required [83–86]. DL-based segmentation algorithms have shown feasibility for automated quantification of tricuspid inflow and annular motion, although these applications remain largely investigational [87].

CCT provides high-resolution anatomical imaging critical for pre-procedural planning of transcatheter tricuspid valve interventions [88, 89]. Automated DL-based approaches have been developed to segment the tricuspid annulus, right atrium, and adjacent structures, reducing manual processing time while maintaining accuracy. As transcatheter therapies expand, integration of CT-based automation with echocardiographic AI frameworks may streamline procedural workflows and enable pre-intervention planning [86, 88].

## Pulmonary Valve

### Pulmonary Stenosis and Regurgitation

Pulmonary valve (PV) disease is relatively uncommon compared with other VHD, and it is most often related to congenital heart disease (CHD) such as Tetralogy of Fallot (TOF), or secondary to pulmonary hypertension [90–94]. Pulmonary stenosis (PS) typically results from congenital valvular or subvalvular obstruction, whereas pulmonary regurgitation (PR) most commonly develops following surgical or transcatheter intervention, or in association with RV outflow tract dilation and elevated pulmonary pressures.

TTE remains the first-line modality for assessing PS and PR, offering Doppler-based quantification of transvalvular gradients and regurgitant flow, while 3D echocardiography provides valuable anatomic detail for procedural planning in CHD. Despite its central role, pulmonary valve imaging is technically challenging due to its anterior position and frequent postsurgical alterations. No dedicated AI models currently exist for PS or PR detection, with most echocardiographic algorithms focused on left-sided lesions; however, AI approaches developed for right-sided imaging, such as automated view classification, 3D RV quantification, and regurgitant jet detection, offer a potentially impactful area for future study.

CMR is a valuable tool in the evaluation of PV disease, providing precise quantification of RV volumes and regurgitant flow. This makes CMR an important tool for surveillance of PR, particularly in TOF, where CMR guides intervention timing. CCT is increasingly used to provide annular and RV outflow tract measurement for transcatheter PV interventions. As percutaneous therapies expand, integrating multimodality imaging and developing AI tools tailored to right-sided and congenital valve disease represent key future directions to improving diagnostic confidence, streamlining surveillance, and guiding intervention timing.

### Current Challenges and Potential Solutions

Despite the substantial potential value of AI in the evaluation and management of VHD, significant challenges remain for its seamless integration into clinical workflows. Most current algorithms are trained on retrospective, single-center datasets that do not capture the full variability of disease phenotypes, imaging quality, or vendor platforms encountered in real-world practice. As a result, algorithmic bias and performance drift across diverse populations are ongoing concerns, underscoring the need for continual validation, recalibration, and post-deployment surveillance.

A critical limitation of many models is explainability. DL models often operate as “black boxes,” producing accurate results without transparent reasoning pathways [95]. This lack of interpretability can undermine clinician confidence and complicate clinical decision-making, especially when outputs influence procedural eligibility or timing of valve replacement. Efforts to build explainable AI frameworks that visually identify salient image regions or provide confidence estimates are essential to foster physician understanding and trust.

Provider trust and workflow implications also shape adoption. Clinicians and sonographers must view AI systems as tools that complement, not replace, their expertise. However, automation could paradoxically increase workload if cardiologists must review a greater number of AI-flagged studies or if faster acquisition expectations strain sonographers. These dynamics call for proactive dialogue among professional societies, hospital leadership, and payers to ensure that reimbursement models evolve alongside technology in ways that preserve workforce sustainability and quality of care.

Ethical and regulatory considerations will play an equally central role in determining the pace of adoption. The need for transparency, bias mitigation, and human oversight is paramount, particularly as AI-derived measurements begin to affect treatment decisions and procedural candidacy. Collaboration between clinicians, engineers, and regulatory agencies will be essential to establish validation frameworks that prioritize both safety and equity. The U.S. Food and Drug Administration currently regulates AI technology in healthcare, and must grapple with privacy, cybersecurity, and post-marketing accountability challenges [95, 96].

Ultimately, the path forward will depend on close collaboration among clinicians, engineers, data scientists, payers, and patient advocates. Standardizing ethical AI development [97–99], incorporating AI education into cardiology training [100], mandating transparent performance reporting, and maintaining human accountability in all decision-making loops will help ensure that AI integration enhances, rather than disrupts, clinical care.

### Future Directions

Future work should focus on expanding AI beyond task- or modality-specific classification to multimodal, clinically integrated systems combining clinical, echocardiography, CCT, CMR data for unified diagnostic and prognostic assessment. While current systems have shown promise in evaluating diseases of multiple valves, performance in polyvalvular disease remains uncertain [22, 39]. Complex hemodynamic interactions may obscure accurate grading; for example, in MR with concomitant AS, the severe MR jet may cause underestimation of the AS gradient, influencing treatment decisions [101, 102]. This underscores a key gap where AI could integrate discordant data from multi-modality imaging to improve severity assessment of polyvalvular disease.

The field of AI in VHD has also reached an inflection point, shifting from algorithm development toward implementation and evaluation. Large-scale, prospective, and federated datasets incorporating diverse populations and imaging vendors will be crucial to ensure model generalizability and equity. Yet as performance matures, implementation science will be equally important: studying how clinicians and sonographers interact with AI tools, understanding provider trust, optimizing interpretability, and rigorously evaluating the cost-effectiveness of AI-guided solutions. Prospective implementation trials, including randomized controlled studies, are needed to bridge the gap between retrospective validation and real-world impact. Ultimately, embedding AI within clinical workflows, reporting systems, and imaging hardware could transform imaging from manual interpretation to precision-guided, data-driven decision-making.

## Conclusions

Artificial intelligence is poised to transform valvular heart disease imaging by enhancing diagnostic precision, efficiency, and standardization across modalities. However, its true value will depend not only on algorithmic performance but also on thoughtful implementation, ethical oversight, and clinician engagement. Through multidisciplinary collaboration, rigorous validation with prospective evaluation, and thoughtful implementation, AI can evolve from an experimental adjunct to a trusted clinical partner, augmenting human expertise while improving patient outcomes. As integration expands, AI-driven imaging has the potential to democratize access to expert-level diagnostics and usher in a new era of precision cardiovascular care.

## Key References

- Holste G, Oikonomou EK, Tokodi M, Kovács A, Wang Z, Khera R. Complete AI-Enabled Echocardiography Interpretation With Multitask Deep Learning. JAMA. 2025 July 22;334(4):306–18.
  - ○ The PanEcho platform is an open-source model which is well validated (internal, external, and in point-of-care settings), with full generation of complete TTE reports.
- Jain SS, Elias P, Poterucha T, Randazzo M, Lopez Jimenez F, Khera R, et al. Artificial Intelligence in Cardiovascular Care-Part 2: Applications: JACC Review Topic of the Week. J Am Coll Cardiol. 2024 June 18;83(24):2487–96.
  - ○ This two-part overview of AI and cardiovascular disease provides a great foundation for future directions and implementation hurdles.

**Table 1:** Comparison of selected complete-TTE interpretation models

	Training size	Validation size	Test size	Aortic valve		Mitral valve		Tricuspid valve	
				Stenosis	Regurgitation	Stenosis	Regurgitation	Stenosis	Regurgitation
PanEcho (22) §‡	999,727 TTE videos from 25,130 studies of 4588 patients.	Internal: 5,130 studies from 4,588 patients External: 18,862 videos from 944 studies of 831 patients.	None	AUC 0.98** (internal); 1.00** (external)	AUC 0.92* (internal); 0.85* (external)	AUC 0.96 (internal); 1.00 (external)	AUC 0.91* (internal); 0.92* (external)	-	AUC 0.90* (internal); 0.91* (external)
EchoPrime (39) ‡	11,984,170 videos from 272,256 studies of 107,663 patients.	25,167 videos from 565 studies of 250 patients.	Internal: 114,831 videos from 2,621 studies across 1,000 patients External: 89,638 videos from 1,792 TTE of 1,792 patients.	AUC 0.98* (internal); 0.96* (external)	AUC 0.88* (internal); 0.89* (external)	AUC 0.96* (internal); 0.92* (external)	AUC 0.92* (internal), 0.95* (external)	-	AUC 0.95* (internal); 0.95* (external)
DELINEATE-Regurgitation (28) §	Internal: 755,283 color Doppler videos of 43,781 TTEs in 26,724 patients.	Internal: 151,344 color Doppler videos from 8,891 TTEs of 8,891 patients.	Internal: 152,543 Doppler videos of 8,987 TTEs of 8,987 patients. External: 144,810 Doppler videos of 10,001 TTEs of 10,001 patients.	-	AUC 0.992* (internal); 0.980* (external)	-	AUC 0.982* (internal); 0.978* (external)	-	AUC 0.976* (internal); 0.95* (external)
Yang et al. (30) §	Retrospective dataset: 1,335 VHD TTEs; 919 normal TTEs Trained (*n* = 1,335)	Retrospective dataset: 311 VHD TTEs; 295 normal TTEs	Retrospective dataset: 434 VHD TTEs, 197 normal TTEs Prospective dataset: 1,374 TTEs	AUC 0.97***	AUC 0.90***	AUC 0.99***	AUC 0.88***	-	-
Us2.AI †§	Unknown training size. AS (33,101); MR (34); TR (80): 1,600 patients	MR: 305 patients	MR: 133 patients	Sensitivity 47.2%**; Specificity 98.1%**	-	-	Accuracy 0.97*	-	Accuracy 0.91*

*Moderate or greater, **Severe, ***mild or greater; VHD = valvular heart disease; TTE = transthoracic echocardiography; † FDA approved; ‡ open-source; § supervised

## Data Availability

The review is based on publicly available data. All data relevant to the study are included in the manuscript.
